# Self-Determination Through Technology: Understanding Physical Activity Engagement for Older Adults

**DOI:** 10.1093/geroni/igaa057.3118

**Published:** 2020-12-16

**Authors:** Lyndsie Koon, Sean Mullen, Wendy Rogers

**Affiliations:** 1 University of Kansas, Lawrence, Kansas, United States; 2 University of Illinois at Urbana-Champaign, Urbana, Illinois, United States; 3 University of Illinois at Urbana-Champaign, Champaign, Illinois, United States

## Abstract

Despite the known health benefits of exercise, only 30% of older adults (65-75 years) and 18.5% (85 years+) meet the recommendations for exercise. Barriers include difficulty accessing facilities, and lack of motivation and social support. Research results indicate that exercise adoption and adherence is higher among older adults when basic psychological needs are met. Technologies (e.g., exergames, activity trackers) have the potential to satisfy the three basic needs as indicated by the Self-Determination Theory. Technology may satisfy a user’s need for autonomy by offering different activities to choose from (biking versus resistance training) or intensity and duration options. They may promote competence by allowing for individualized goal setting and tracking. Technologies have the potential to promote relatedness through virtual instruction, subsequently removing the accessibility barrier. The application of this theory provides design guidelines for exercise technologies and a greater understanding of how technology may motivate exercise behavior for older adults

